# USING MOBILE TECHNOLOGY TO ELUCIDATE PSYCHOSOCIAL AND COGNITIVE FUNCTION IN OLDER ADULTS

**DOI:** 10.1093/geroni/igz038.2982

**Published:** 2019-11-08

**Authors:** Nelson A Roque, Jinshil Hyun, Stacey B Scott

**Affiliations:** 1 Pennsylvania State University, Center for Healthy Aging, University Park, Pennsylvania, United States; 2 Albert Einstein College of Medicine, Bronx, New York, United States; 3 Stony Brook University, Stony Brook, New York, United States

## Abstract

Ambulatory methods (AM) improve the reliability and ecological validity of cognitive assessments, and help to elucidate psychological influences through concurrent reports of pain, stress, and other psychosocial outcomes. Ecological momentary assessment (EMA) involves sampling of daily experiences in natural settings, including completing cognitive assessments, and answering questions related to, for example, social interactions and sleep. The purpose of this symposium is to present innovative methods and results, exploring questions at the intersection of intensive longitudinal data collection, cognition, and psychosocial influences, using data from two EMA studies, the Einstein Aging Study (EAS) and the Effects of Stress on Cognitive Aging, Physiology, and Emotion (ESCAPE) Study. The EAS (ages >= 70) and ESCAPE (ages 25 - 65) protocols, ask participants to complete an annual 14-day EMA measurement burst. A unique value of these methods is the ability to explore effects from moment-to-moment (or day-to-day; within-person effects) as we will present. We will also contrast these with conventional analyses of between-person differences, typical of clinic and in-person studies. Dickens (using ESCAPE data) examines end-of-day perceived stress and anticipation of next-day stress in predicting sleep quality. Hyun and colleagues (using EAS data) discuss the effects of affectionate physical touch on mitigating pain and emotional distress. Using a model-based cluster analysis approach (with EAS data), Roque unpacks differences in psychosocial factors, as a function of cognitive status risk groups. Stacey Scott will discuss these papers in the context of using ambulatory methods to improve the characterization of risk status in older adults.

